# Utilizing conditional generative adversarial network to generate head MRA based on nonvascular sequences: comparative study of single-modality and multi-modality methods

**DOI:** 10.3389/fneur.2026.1857271

**Published:** 2026-07-06

**Authors:** Xinyu Song, Lei Xiang, Ping Wang, Chao Zhu, Zhongzheng Cao, Xiaoer Wei, Tianen Yu, Tao Zhou, Yuehua Li

**Affiliations:** 1 Institute of Diagnostic and Interventional Radiology, Shanghai Sixth People's Hospital Affiliated to Shanghai Jiao Tong University School of Medicine, Shanghai, China; 2 ShanghaiTech University, Shanghai, China; 3 Nanjing University of Science and Technology, Nanjing, China

**Keywords:** diagnosis, generative adversarial network, magnetic resonance angiography, multimodality imaging, time-of-flight

## Abstract

**Objective:**

To develop a deep learning model synthesizing head MRA images from the preferred nonvascular sequences (T1W, T2W, and FLAIR) and evaluate the diagnostic performance of synthetic MRA (syn-MRA).

**Methods:**

This retrospective study included geriatric inpatients who underwent multimodal MRI (including MRA and nonvascular sequences) at our institution between January 2022 and March 2023. Three single-modality conditional generative adversarial network (cGAN) models (T1W, T2W, and FLAIR-based) and one multi-modality model (MIX model) were constructed. Quantitative metrics were used to evaluate image quality. Two radiologists independently assessed image visual quality using Likert scales. Two senior neuroradiologists evaluated structured reports and diagnostic confidence.

**Results:**

This study ultimately included 140 patients: 98 training, 14 validation, 28 testing. The MIX model outperformed single-modality models (test set: structural similarity index measure 0.880, peak signal-to-noise ratio 33.18 dB, root mean squared error 0.022). Syn-MRA from all models demonstrated significantly higher signal-to-noise ratio and contrast-to-noise ratio compared to real MRA (*p* < 0.001), improved vascular signal uniformity (*p* > 0.05), but more pronounced venous contamination (*p* < 0.001). The MIX model achieved comparable overall image quality (median: 5 vs. 5, *p* = 0.344), vessel sharpness (median: 5 vs. 5, *p* = 0.145), and diagnostic confidence (median: 5 vs. 5, *p* = 0.102) relative to real MRA. MIX model syn-MRA showed 92.2% diagnostic accuracy (vessel level).

**Conclusion:**

The MIX model outperformed single-modality models, with image quality and diagnostic performance comparable to real MRA.

## 1 Introduction

Time-of-flight magnetic resonance angiography (TOF-MRA) is a widely used technique for cerebrovascular imaging (1), offering high spatial resolution, multi-angle vascular visualization, and eliminating the need for iodinated contrast agents. It remains the primary modality for detecting vascular anomalies, stenoses, and aneurysms (2–4). Despite its clinical utility, clinicians frequently deferred TOF-MRA until after detecting abnormalities in nonvascular sequences owing to prolonged acquisition times, potentially leading to missed incidental vascular pathologies. Prolonged scanning durations not only increase motion artifact risks (5, 6), but may also lead to incomplete examinations. Current multi-modality imaging techniques allow simultaneous acquisition of multiple contrast-weighted images through a single acquisition (7, 8), significantly reducing examination time compared to conventional protocols. However, MRA still requires additional acquisition.

Generative neural network models have been extensively applied to medical imaging tasks (9), with the generative adversarial network (GAN) architecture demonstrating significant success in vessel segmentation (10), image enhancement (11), and cross-modal synthesis (12). These advancements have spurred growing interest in intracranial MRA synthesis. Current intracranial MRA synthesis approaches can be categorized into two types based on input modalities: (1) Vascular-based methods utilized angiographic data, exemplified by You et al. (13), who employed cycleGAN for 4D-to-3D MRA reconstruction; (2) Nonvascular-based approaches leveraged conventional nonvascular sequences, such as synthesizing MRA from T1-weighted (T1W) and T2-weighted (T2W) images (14) or integrating multi-modality nonvascular images (15). However, nonvascular sequences are routinely acquired first in clinical practice, making nonvascular-based synthesis more promising for clinical application. In addition, nonvascular-based MRA synthesis studies predominantly employed multi-modality inputs, yet systematic comparisons between single-modality and multi-modality approaches remain unexplored. Meanwhile, validation efforts in these studies had focused on single-disease cohorts, such as large vessel occlusion or aneurysm, failing to comprehensively represent the overall diagnostic performance of synthetic MRA (syn-MRA).

In this study, we propose a deep learning framework for synthesizing high-quality MRA images from clinically accessible T1W, T2W, and fluid-attenuated inversion recovery (FLAIR) sequences. The objectives of this study were threefold: (1) to develop and validate a conditional GAN (cGAN) model for MRA synthesis, (2) to perform a comparative analysis of MRA generation performance between single-modality (T1W/T2W/FLAIR) and multi-modality approaches, and (3) to assess clinical applicability through structured diagnostic reports evaluating syn-MRA performance.

## 2 Methods

This retrospective study was approved by the Ethics Committee of Shanghai Sixth People’s Hospital (NO. 2024-KY-176[K]). The requirement for written informed consent was waived due to the use of anonymized historical database.

### 2.1 Data acquisition

**Figure 1:**
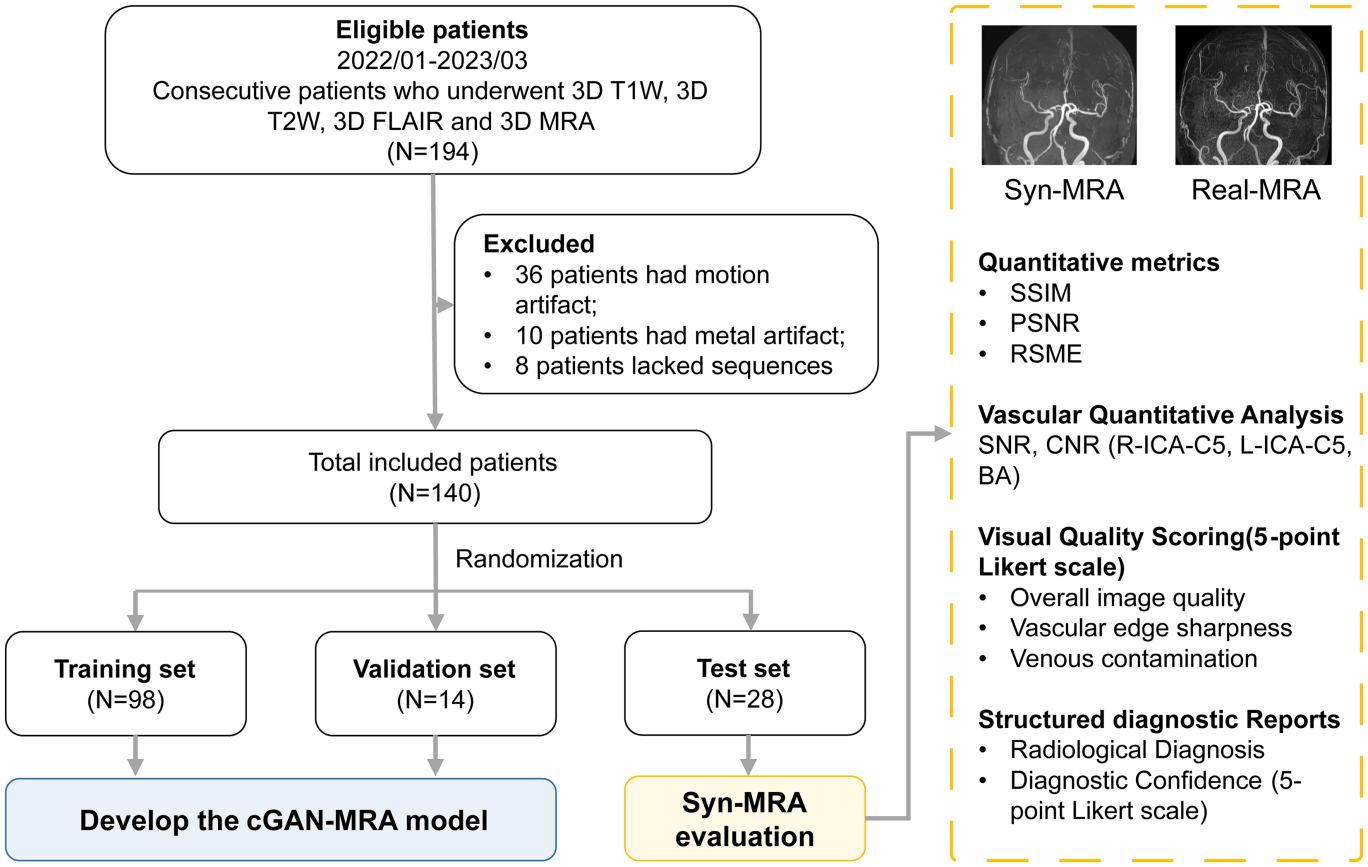
Data flow diagram for model construction and evaluation. T1W = T1-weighted, T2W = T2-weighted, FLAIR = fluid attenuated inversion recovery, MRA = magnetic resonance angiography, cGAN = conditional generative adversarial network, syn-MRA = synthetic MRA, SSIM = structural similarity index measure, PSNR = peak signal-to-noise ratio, RMSE = root mean square error, SNR = signal-to-noise ratio, CNR = contrast-to-noise ratio, R-ICA = right internal carotid artery, L-ICA = left internal carotid artery, C5 = clinoid segment, BA = basilar artery.

This study cohort comprised 194 inpatients from the Department of Geriatrics who underwent standardized multimodal brain MRI between January 2022 and March 2023 using 3T scanners (Prisma/Skyra, Siemens Healthcare) in our research center. The imaging sequences included 3D T1W, 3D T2W, 3D FLAIR, and 3D TOF MRA. Full detailed imaging protocols are provided in Supplementary Table S1. After excluding images with motion artifacts (*n* = 36), metal artifacts (*n* = 10) and those lacking sequences (*n* = 8), 140 patient images remained. These patients were allocated through randomization into training (*n* = 98), validation (*n* = 14), and test sets (*n* = 28) using a 7:1:2 ratio. The training and validation set were used for model development, while the test set was used for quantitative and clinical evaluation of the model (Figure 1).

### 2.2 Model establishment

**Figure 2:**
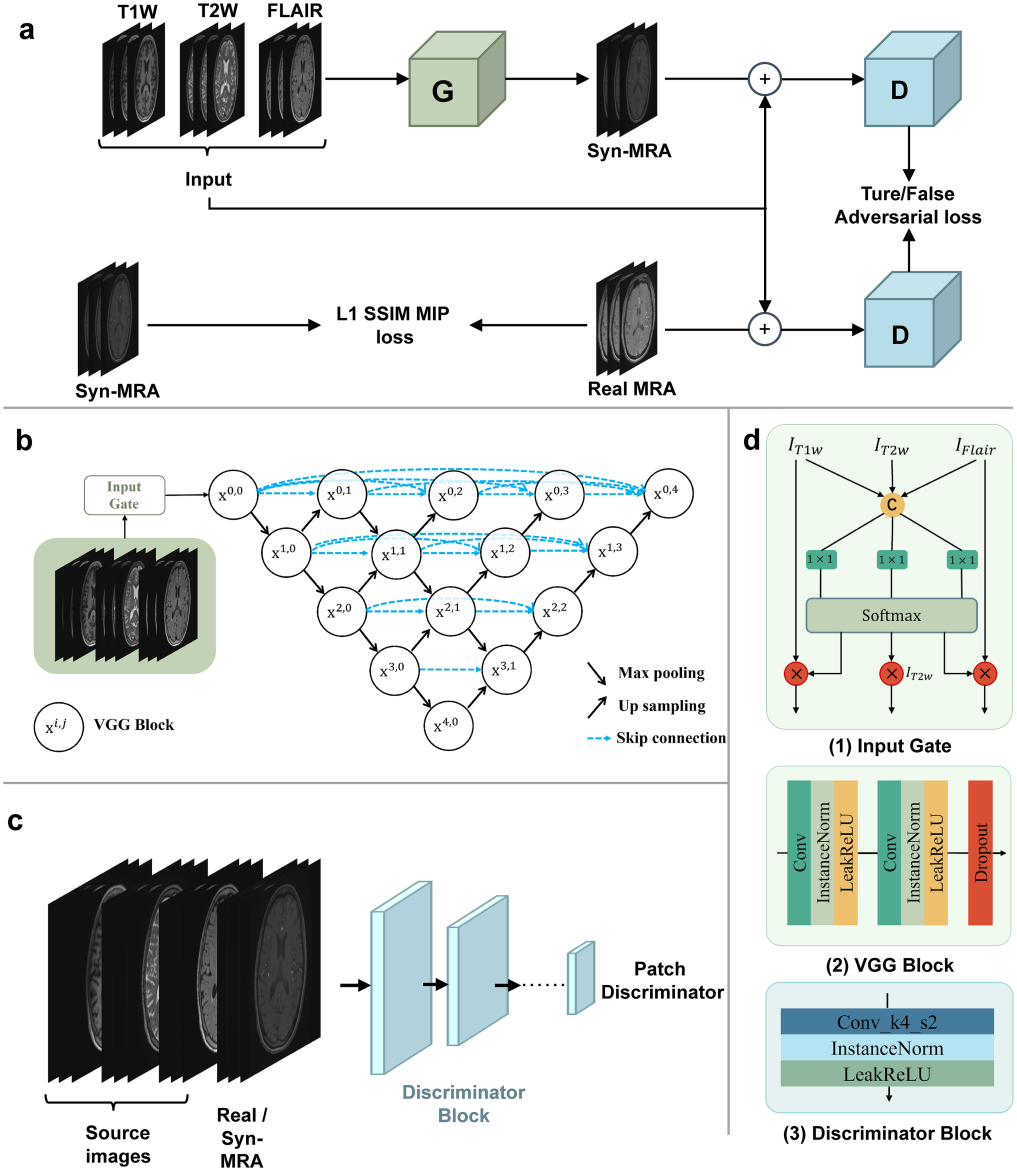
Network architecture of the conditional generative adversarial network magnetic resonance angiography (cGAN-MRA) model. **(a)** The cGAN model’s framework. **(b)** Generator structure based on the U-Net++. **(c)** Patch-based discriminator. **(d)** The structures of the modules used models: (1) the convolutional gating module before the input of Unet++, (2) the Unet++ base VGG module, (3) the discriminator base module. T1W = T1-weighted, T2W = T2-weighted, FLAIR = fluid attenuated inversion recovery, syn-MRA = synthetic magnetic resonance angiography, MIP = maximum intensity projection; SSIM = structural similarity index measure; VGG = visual geometry group.

To generate syn-MRA images, we developed a conditional generative adversarial network (16) magnetic resonance angiography (cGAN-MRA) model. The cGAN consists of two independent modules: the generator and the discriminator. The underlying architecture of the generator is Unet++ (17). The discriminator adopts the patch-based approach, which evaluates local patches of the image rather than the entire image. To avoid stripe artifacts along non-imaging planes in 3D images, we utilized a 2.5D input setting comprising 5 adjacent 2D slices simultaneously during training, and the outputs were averaged based on their overlapping times. Since clinical vascular diagnosis prefers maximum intensity projection (MIP) images, we designed a loss function (MIP loss) sensitive to maximum intensity values to enhance the control of fine details in the syn-MRA images. Imaging preprocessing and details of model construction are provided in Supplementary Material 1. The structure of the cGAN-MRA model is shown in Figure 2.

To compare single- and multi-modality models, this study constructed three single-modality generative network models (T1W model, T2W model, and FLAIR model) and one multi-modality generative network model (MIX model) based on the cGAN-MRA framework. The T1W model, T2W model, and FLAIR model used T1W, T2W, and FLAIR images as inputs respectively, with TOF-MRA as the ground truth for single-modality model development. The MIX model utilized all images as inputs for multi-modality model construction. To optimally fuse multi-modality inputs, a convolutional gating module was introduced before the U-Net++ generator. It dynamically emphasizes informative cross-modal features by directly applying spatial attention weights—derived via convolutional and sigmoid layers—to the concatenated inputs. To assess the performance of the four trained cGAN-MRA models, the Structural Similarity Index (SSIM), Peak Signal-to-Noise Ratio (PSNR), and Root Mean Square Error (RMSE) were calculated between the synthesized outputs and the ground truth on both TOF slices and axial MIP images. The detailed calculation formulas are provided in Supplementary Material 2.

### 2.3 Vascular quantitative analysis

Two radiologists (R1, 9 years’ experience; R2, 10 years’ experience), blinded to image origins, independently measured signal-to-noise ratio (SNR) and contrast-to-noise ratio (CNR) for test set images, including real MRA and syn-MRA outputs from four models (28 patients × 5 conditions). Regions of interest (ROIs) were manually delineated on native MRA images in the middle of the right internal carotid artery C5 segment (ICA-C5), left ICA-C5, and basilar artery (BA). Background signals were obtained from brainstem tissue at identical slice levels with same size ROIs (18). The final measurements derived from averaged values of both observers. Formulas are shown in Supplementary Material 2. Measurements were performed using a commercial DICOM viewer (U-Viewer, version R001.0.0.2).

### 2.4 Visual quality scoring

R1 and R2 independently evaluated the visual quality of test set images using 5-point Likert scales on MIP reconstructions. The scoring criteria comprised three domains: Overall image quality (5 = Excellent, 4 = Good, 3 = Moderate, 2 = Poor, 1 = Nondiagnostic), Vascular edge sharpness (5 = Satisfyingly sharp, 4 = Moderately sharp, 3 = A little sharp, 2 = Not sharp, 1 = Nondiagnostic), Venous contamination (5 = None, 4 = Mild, 3 = Moderate, 2 = Severe, 1 = Nondiagnostic). Detailed scoring criteria are provided in Supplementary Table S2. For cases with interobserver discrepancy, consensus was achieved through joint evaluation with a senior neuroradiologist (R3, 17 years of neuroradiology experience).

### 2.5 Structured diagnostic reports

Two senior neuroradiologists (R3; R4, 22 years of neuroradiology experience) evaluated real MRA and syn-MRA images using a structured reporting system developed in-house (https://rad.cnradiology.org). Assessments were performed on anonymized source images and MIP reconstructions. Diagnostic evaluation focused on major cerebral arteries: ICAs, middle cerebral arteries (MCAs), anterior cerebral arteries (ACAs), vertebral arteries (VAs), BA, and posterior cerebral arteries (PCAs). Small branch vessels were excluded as main trunk pathologies hold greater clinical significance and demonstrated higher interobserver agreement. Diagnostic categories included: (a) arterial stenosis, defined as luminal narrowing >50% (including occlusions); (b) aneurysms, reported as present/absent without subclassification or size measurement; (c) vascular dysplasia, encompassing agenesis, hypoplasia, fenestrations, and other developmental variants; (d) normal artery, absence of detectable pathology. Diagnostic performance evaluation was categorized into patient-level and vessel-level analyses. Patient-level accuracy required complete concordance with the ground truth diagnosis, while vessel-level accuracy assessed agreement for individual vessels. Both radiologists performed diagnostic confidence scoring using a 5-point scale (5 = definite). In cases of disagreement, confirmation was achieved through joint image review.

### 2.6 Statistical analysis

The normality of continuous variables was assessed using the Shapiro–Wilk test. Normally distributed data were presented as mean ± standard deviation, while non-normally distributed data were expressed as median (interquartile range [IQR]). Categorical variables were reported as frequencies with percentages. Paired t-tests were employed for comparing quantitative metrics. Interobserver agreement was quantified using intraclass correlation coefficients (ICC) and weighted kappa coefficients (*κ*), with values >0.800 indicating excellent agreement. Diagnostic performance was evaluated using sensitivity, specificity, positive predictive value (PPV), negative predictive value (NPV), and accuracy, with 95% confidence intervals (CI) calculated via Wilson’s score method. *p* < 0.05 defined statistical significance. All analyses and visualizations were conducted using Python (version 3.12.4), GraphPad Prism (version 10.1.2), and SPSS (version 27.0.1.0).

## 3 Results

### 3.1 Patients characteristics

This study ultimately included 140 patient images. The cGAN-MRA model was developed using a training set of 98 patients (36.7% male; mean age, 68 ± 8 years) and a validation set of 14 patients (42.9% male; mean age, 65 ± 8 years). The independent test set comprised 28 patients (39.3% male; mean age, 71 ± 8 years) (Supplementary Table S3).

### 3.2 Quantitative evaluation of models

Quantitative comparisons across the four models demonstrated superior performance of the MIX model compared to single-modality approaches in both syn-source-MRA and syn-MIP-MRA (Table 1). For syn-source-MRA in the test set, the MIX model achieved SSIM of 0.880 ± 0.032, PSNR of 33.18 ± 1.21 dB, and RMSE of 0.022 ± 0.003. Corresponding metrics for syn-MIP-MRA were SSIM = 0.791 ± 0.043, PSNR = 26.60 ± 1.45 dB, and RMSE = 0.047 ± 0.008. The ablation study demonstrated that incorporating the MIP loss consistently improves the quantitative parameters of the syn-MIP-MRA images across all models (Supplementary Table S4).

**Table 1:** Quantitative comparisons of synthetic MRA images generated by single-modality or multi-modality models.

Models	Validation set			Test set		
	SSIM	PSNR	RMSE	SSIM	PSNR	RMSE
syn-source-MRA						
T1W	0.860 ± 0.333	32.46 ± 1.27	0.024 ± 0.004	0.865 ± 0.031	32.15 ± 1.02	0.025 ± 0.003
T2W	0.860 ± 0.034	32.55 ± 1.26	0.024 ± 0.004	0.863 ± 0.035	32.46 ± 1.31	0.024 ± 0.004
FLAIR	0.855 ± 0.032	32.33 ± 1.16	0.024 ± 0.004	0.859 ± 0.032	32.19 ± 1.04	0.025 ± 0.003
MIX	**0.877 ± 0.035**	**33.33 ± 1.48**	**0.022 ± 0.004**	**0.880 ± 0.032**	**33.18 ± 1.21**	**0.022 ± 0.003**
syn-MIP-MRA						
T1W	0.754 ± 0.060	25.62 ± 1.91	0.054 ± 0.012	0.761 ± 0.040	25.87 ± 1.47	0.052 ± 0.009
T2W	0.770 ± 0.058	26.16 ± 1.75	0.050 ± 0.011	0.773 ± 0.043	26.19 ± 1.68	0.050 ± 0.010
FLAIR	0.759 ± 0.056	25.67 ± 1.72	0.053 ± 0.011	0.763 ± 0.042	25.79 ± 1.69	0.052 ± 0.010
MIX	**0.785 ± 0.059**	**26.32 ± 1.91**	**0.049 ± 0.012**	**0.791 ± 0.043**	**26.60 ± 1.45**	**0.047 ± 0.008**

The results are presented as mean ± standard deviation, and the best measures are highlighted in bold. The higher PSNR and SSIM, the lower the RMSE values indicate better performance. T1W, T2W, FLALR represent single-modality models; MIX represents multi-modality model. syn-source-MRA = synthetic source magnetic resonance angiography, syn-MIP-MRA = synthetic maximum intensity projection magnetic resonance angiography, T1W = T1-weighted, T2W = T2-weighted, FLAIR = fluid attenuated inversion recovery, SSIM = structural similarity index measure, PSNR = peak signal-to-noise ratio, RMSE = root mean square error.

### 3.3 Vascular quantitative analysis

As shown in Table 2, the syn-MRA images from all models demonstrated significantly higher mean SNR than real MRA images (*p* < 0.001), with analogous findings observed for CNR values (*p* < 0.001). While BA exhibited higher mean SNR than ICA-C5 in real MRA images, syn-MRA images from all models reduced this disparity (Supplementary Figure S1). The mean ratio of BA/ICA-C5 SNR (r-SNR) was lower for syn-MRA than for real MRA, though this difference lacked statistical significance (*p* > 0.05). Similarly, no significant reduction was observed in CNR ratio comparisons (*p* > 0.05). Excellent interobserver agreement was achieved for most metrics (ICC > 0.800), particularly for images generated by the MIX model (Supplementary Table S5).

**Table 2:** Vascular quantitative analysis and visual quality scoring of syn-MRA.

Quantitative	Real MRA	Syn-MRA(T1W)	Syn-MRA(T2W)	Syn-MRA(FLAIR)	Syn-MRA(MIX)	*P*-value^*^	*P*-value^†^	*P*-value^‡^	*P*-value^§^
SNR									
R-ICA-C5	23 ± 8	309 ± 42	272 ± 49	250 ± 45	299 ± 56	<0.001	<0.001	<0.001	<0.001
L-ICA-C5	25 ± 8	334 ± 53	248 ± 51	269 ± 44	329 ± 61	<0.001	<0.001	<0.001	<0.001
BA	35 ± 8	398 ± 49	353 ± 51	306 ± 60	406 ± 75	<0.001	<0.001	<0.001	<0.001
r-SNR^1^	1.6 ± 0.5	1.3 ± 0.1	1.3 ± 0.3	1.3 ± 0.4	1.4 ± 0.2	0.067	0.112	0.099	0.150
r-SNR^2^	1.5 ± 0.4	1.3 ± 0.1	1.3 ± 0.4	1.2 ± 0.3	1.2 ± 0.2	0.199	0.257	0.059	0.142
CNR									
R-ICA-C5	19 ± 8	216 ± 34	184 ± 40	167 ± 40	210 ± 45	<0.001	<0.001	<0.001	<0.001
L-ICA-C5	21 ± 8	242 ± 44	197 ± 44	186 ± 40	240 ± 51	<0.001	<0.001	<0.001	<0.001
BA	31 ± 7	307 ± 39	265 ± 47	223 ± 55	317 ± 61	<0.001	<0.001	<0.001	<0.001
r-CNR^1^	1.9 ± 0.8	1.4 ± 0.2	1.5 ± 0.6	1.4 ± 0.7	1.5 ± 0.3	0.121	0.226	0.219	0.226
r-CNR^2^	1.6 ± 0.5	1.4 ± 0.2	1.5 ± 0.7	1.2 ± 0.4	1.3 ± 0.3	0.329	0.592	0.098	0.207
Visual Quality Scoring									
Overall image quality	5(4–5)	3(3–4)	3(3–4)	2(2–2)	5(4–5)	<0.001	<0.001	<0.001	0.344
Vascular edge sharpness	5(4.3–5)	3(3–4)	3.5(3–4)	3(2–3)	5(4–5)	<0.001	<0.001	<0.001	0.145
Venous contamination	5(5–5)	3(3–4)	3(2–3)	3(2–3)	3(3–3.8)	<0.001	<0.001	<0.001	<0.001
Diagnostic Confidence	5(5–5)	3(2–3)	3(2–4)	2(2–2)	5(4–5)	<0.001	<0.001	<0.001	0.102

Data are presented as mean ± standard deviation or median (IQR). syn-MRA = synthetic magnetic resonance angiography; T1W = T1-weighted, T2W = T2-weighted, FLAIR = fluid attenuated inversion recovery, SNR = signal-to-noise ratio, CNR = contrast-to-noise ratio, R-ICA = right internal carotid artery, L-ICA = left internal carotid artery, C5 = clinoid segment, BA = basilar artery. r-SNR^1^ and r-CNR^1^ represent the ratio of BA to R-ICA-C5; r-SNR^2^ and r-CNR^2^ represent the ratio of BA to L-ICA-C5. ^*^Syn-MRA(T1W) versus Real MRA, ^†^Syn-MRA (T2W) versus Real MRA, ^‡^Syn-MRA (FLAIR) versus Real MRA, ^§^Syn-MRA (MIX) versus Real MRA.

### 3.4 Visual quality evaluation

Across all visual quality assessment categories (Table 2), the MIX model-generated Syn-MRA images demonstrated no significant differences in overall image quality (median, 5 [IQR 4–5] vs. 5 [IQR 4–5]; *p* = 0.344) and vascular edge sharpness (median, 5 [IQR 4–5] vs. 5 [IQR 4.3–5]; *p* = 0.145) compared to real MRA images. In contrast, Syn-MRA from single-modality models showed significantly lower scores than real MRA (*p* < 0.001). All models, including single- and multi-modality approaches, exhibited pronounced venous contamination (*p* < 0.001).

### 3.5 Structured diagnostic reports

Based on structured report results, real MRA images identified arterial stenosis (>50%) in 2 patients (7.1%), aneurysms in 3 patients (10.7%), arterial dysplasia in 9 patients (32.1%), and normal arteries in 15 patients (53.6%).

**Figure 3:**
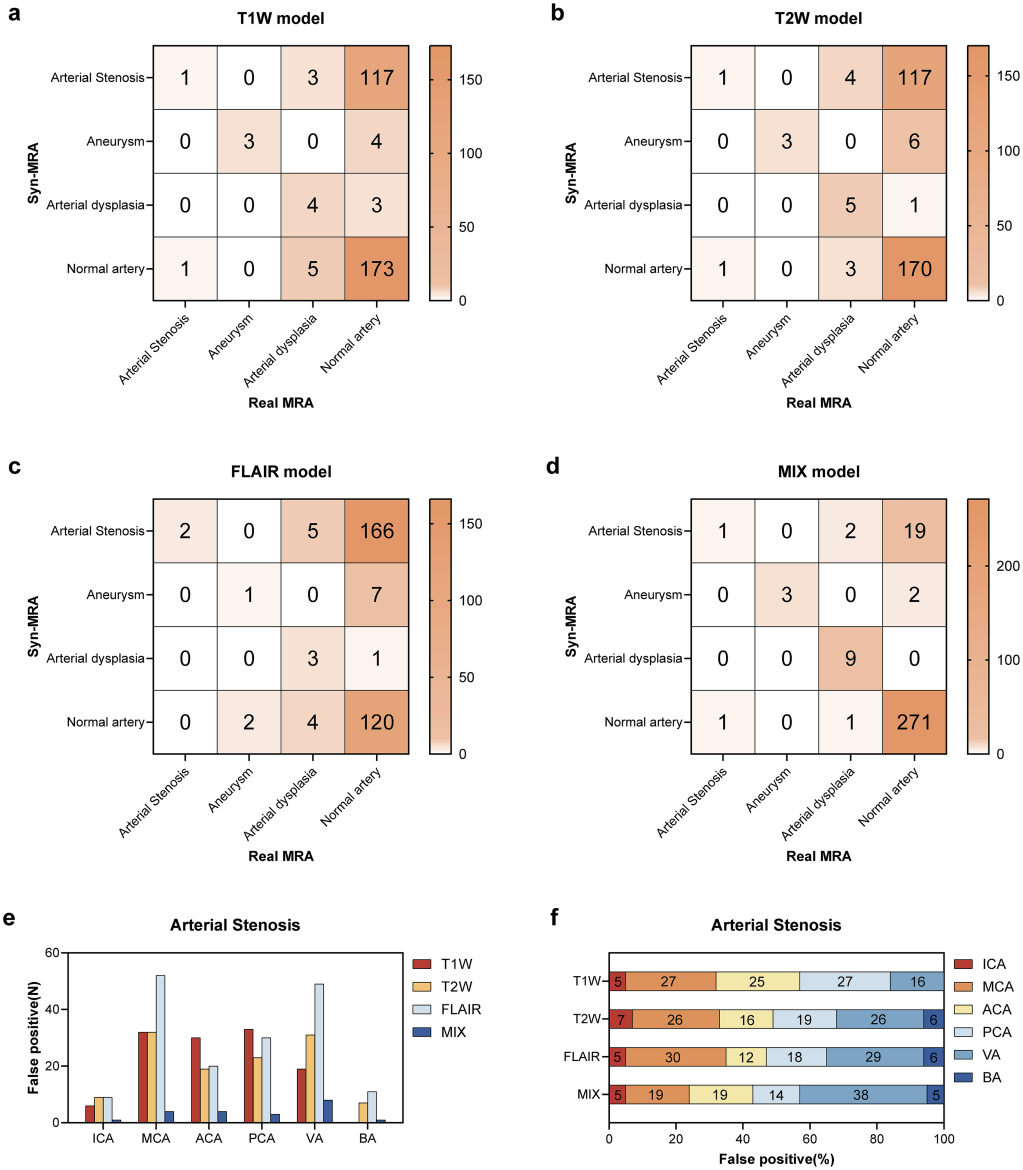
Arterial diagnosis using syn-MRA versus real MRA images in test set. Confusion matrixes **(a–d)** showed that the MIX model-based syn-MRA had the highest diagnostic accuracy and lowest false-positive and false-negative rates compared to all single-modality models. Notably, the highest false-positive rates for arterial stenosis were observed across all models. **(e,f)** Bar charts illustrated the number and proportion of false-positive diagnoses for arterial stenosis across different models. False-positive rates were lowest in the internal carotid artery and basilar artery across all models, while the MIX model had the fewest false-positive cases in all types of vessels. T1W = T1-weighted, T2W = T2-weighted, FLAIR = fluid attenuated inversion recovery, syn-MRA = synthetic magnetic resonance angiography, ICA = internal carotid artery, MCA = middle cerebral artery, ACA = anterior cerebral artery, PCA = posterior cerebral artery, VA = vertebral artery, BA = basilar artery.

At the vessel level, the MIX model-generated syn-MRA images demonstrated superior diagnostic accuracy (92.2%, 284/308 vessels), lowest false-positive rate (7.5%, 23/308), and minimal false-negative rate (0.6%, 2/308) compared to single-modality models. Arterial stenosis accounted for the majority of false positives (6.8%, 21/308). Across all models, the ICAs and BA exhibited the lowest rates of false-positive stenosis, with the MIX model achieving minimal false-positive stenosis rates across all vessel types. Specifically, the MIX model had the highest false-positive stenosis rate in VAs (14.3%, 8/56), whereas single-modality models showed peak false positives in MCAs (Figure 3 and Table 3).

**Table 3:** False-positive arterial stenosis in syn-MRA based on different models in vessel level.

Vessel segments	T1W	T2W	FLAIR	MIX
Total	120/308 (39.0)	121/308 (39.3)	171/308 (55.5)	21/308 (6.8)
ICA	6/56 (10.7)	9/56 (16.1)	9/56 (16.1)	1/56 (1.8)
MCA	32/56 (57.1)	32/56 (57.1)	52/56 (92.9)	4/56 (7.1)
ACA	30/56 (53.6)	19/56 (33.9)	20/56 (35.7)	4/56 (7.1)
PCA	33/56 (58.9)	23/56 (41.1)	30/56 (53.6)	3/56 (5.4)
VA	19/56 (33.9)	31/56 (55.4)	49/56 (87.5)	8/56 (14.3)
BA	0/28 (0.0)	7/28 (25.0)	11/28 (39.3)	1/28 (3.6)

The results are presented as number/total (%). Syn-MRA = synthetic magnetic resonance angiography, T1W = T1-weighted, T2W = T2-weighted, FLAIR = fluid attenuated inversion recovery, ICA = internal carotid artery, MCA = middle cerebral artery, ACA = anterior cerebral artery, PCA = posterior cerebral artery, VA = vertebral artery, BA = basilar artery.

**Figure 4:**
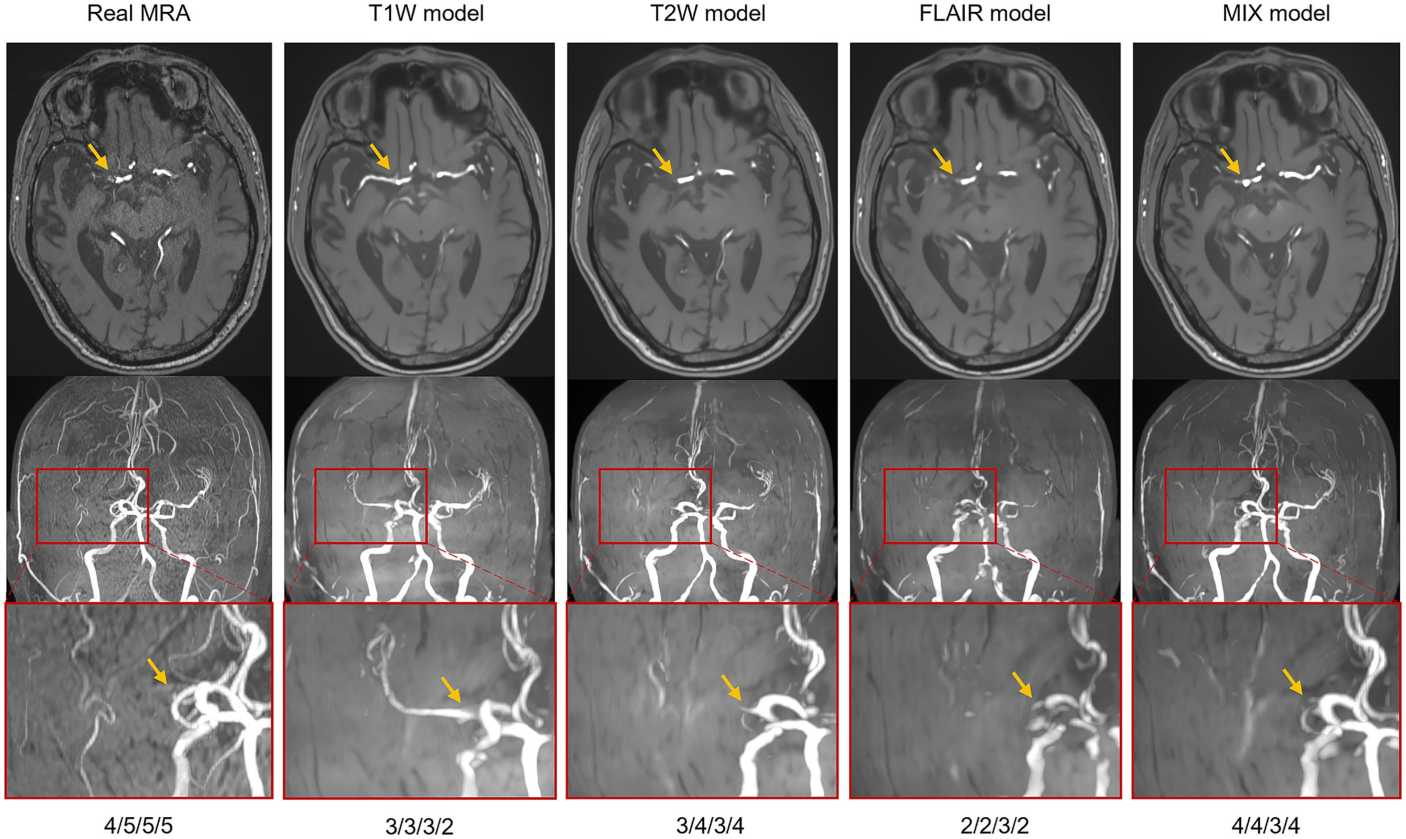
Comparison of synthetic MRA images generated by different Models. Male, 72 years old, real MRA showed occlusion of the right middle cerebral artery. T2W, FLAIR, and MIX model syn-MRA all accurately identified the abnormality, while T1W model syn-MRA showed a false negative at this location (yellow arrow). The red box indicates the magnified image, n/n/n/n means overall image quality/vascular edge sharpness/venous contamination/diagnostic confidence scores. MRA = magnetic resonance angiography, T1W = T1-weighted; T2W = T2-weighted, FLAIR = fluid attenuated inversion recovery.

**Figure 5:**
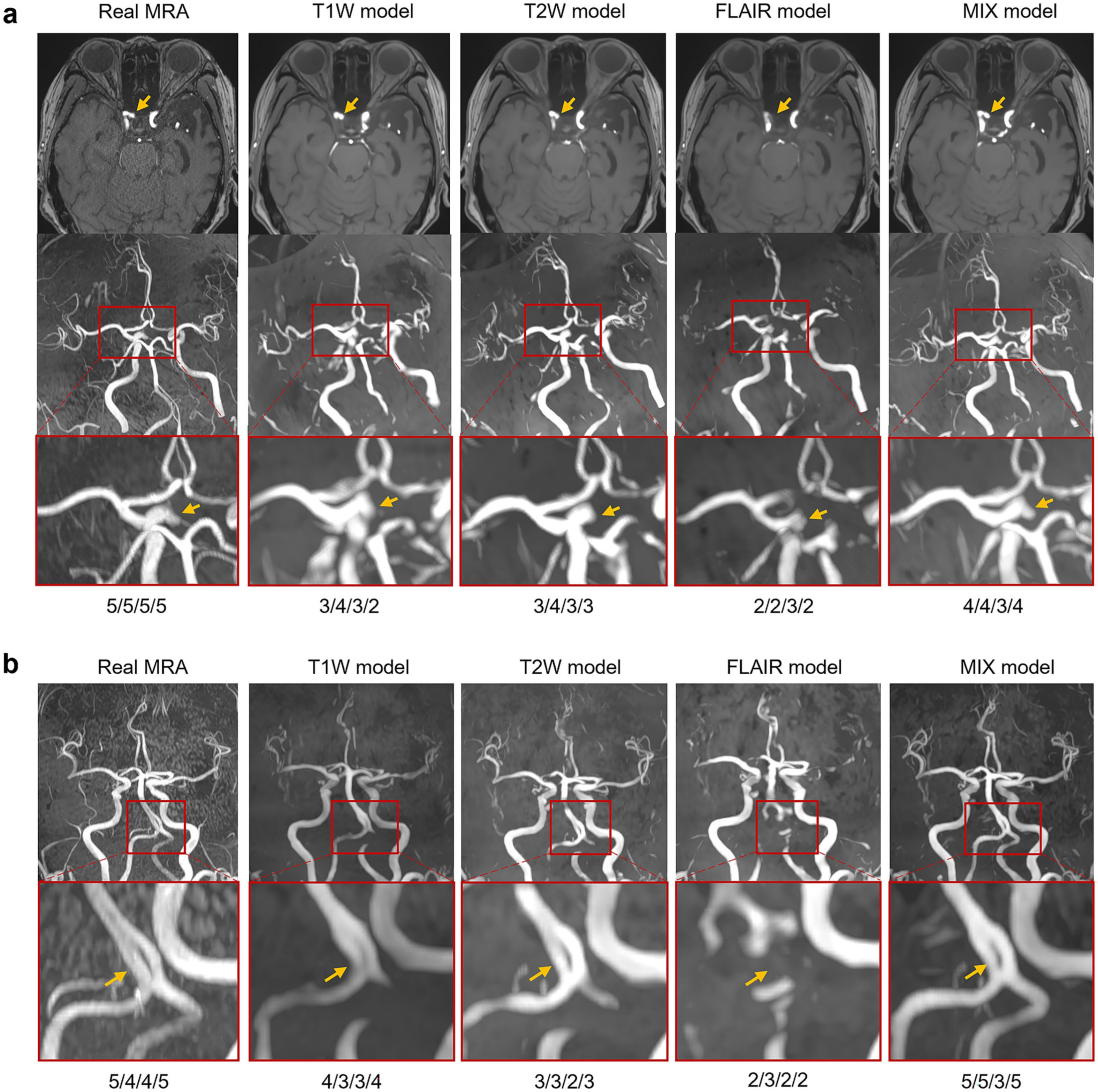
Comparison of synthetic MRA images generated by different Models. **(a)** Female, 76 years old, real MRA demonstrated an aneurysm at the right internal carotid artery C5 segment. T1W, T2W, and MIX model syn-MRA accurately identified the aneurysm, but T2W model showed a smaller size, and FLAIR model failed to display the aneurysm (yellow arrow). **(b)** Male, 81 years old, basilar artery fenestration was observed in the real MRA. While T1W, T2W, and MIX model syn-MRA successfully reconstructed the fenestration, FLAIR model failed (yellow arrow). The red box indicates the magnified image, n/n/n/n means overall image quality/vascular edge sharpness/venous contamination/diagnostic confidence scores. MRA = magnetic resonance angiography, T1W = T1-weighted; T2W = T2-weighted, FLAIR = fluid attenuated inversion recovery.

The MIX model demonstrated optimal diagnostic performance among all models, with excellent agreement against real MRA. At the patient level (Table 4), MIX model syn-MRA achieved an accuracy of 0.786 (95% CI: 0.605–0.898), sensitivity of 0.923 (95% CI: 0.667–0.986), and specificity of 0.666 (95% CI: 0.417–0.848). Vessel-level analysis revealed highest accuracy for the BA (0.964, 95% CI: 0.823–0.994), followed by ICA (0.946, 95% CI: 0.854–0.982), with lowest performance in VA (0.857, 95% CI: 0.743–0.926). Regarding diagnostic confidence, MIX model syn-MRA showed statistically equivalent ratings to real MRA (*p* = 0.102). Representative cases are illustrated in Figures 4, 5 and Supplementary Figure S2.

**Table 4:** Comparisons of diagnostic performance of MIX-model syn-MRA versus real MRA for arterial abnormality in test set.

Stratification level	Sensitivity (95%CI)	Specificity (95%CI)	PPV (95%CI)	NPV (95%CI)	Accuracy (95%CI)
Patient level	0.923 (0.667–0.986)	0.666 (0.417–0.848)	0.706 (0.469–0.867)	0.909 (0.623–0.984)	0.786(0.605–0.898)
Vessel level					
ICA	1.000 (0.439–1.000)	0.943 (0.846–0.981)	0.500 (0.188–0.812)	1.000 (0.929–1.000)	0.946(0.854–0.982)
MCA	1.000 (0.207–1.000)	0.927 (0.827–0.971)	0.200 (0.036–0.624)	1.000 (0.930–1.000)	0.929(0.830–0.972)
ACA	1.000 (0.439–1.000)	0.925 (0.821–0.970)	0.429 (0.158–0.750)	1.000 (0.927–1.000)	0.929(0.830–0.972)
PCA	0.500 (0.150–0.850)	0.942 (0.844–0.980)	0.400 (0.118–0.769)	0.961 (0.868–0.989)	0.911(0.807–0.961)
VA	1.000 (0.439–1.000)	0.849 (0.729–0.921)	0.273 (0.097–0.566)	1.000 (0.921–1.000)	0.857(0.743–0.926)
BA	1.000 (0.207–1.000)	0.963 (0.817–0.993)	0.500 (0.095–0.905)	1.000 (0.871–1.000)	0.964(0.823–0.994)

syn-MRA = synthetic magnetic resonance angiography, ICA = internal carotid artery, MCA = middle cerebral artery, ACA = anterior cerebral artery, PCA = posterior cerebral artery, VA = vertebral artery, BA = basilar artery, CI = confidence internal, PPV = positive predictive value, NPV = negative predictive value.

## 4 Discussion

This study established a cGAN-based head MRA generation model, which utilized single- or multi-modality nonvascular magnetic resonance images as inputs. Compared to real MRA, syn-MRA demonstrated superior performance in reducing image background noise and improving arterial signal-to-noise ratio, while enhancing vascular signal uniformity. In visual quality assessments, multi-modality model-based syn-MRA achieved comparable quality to real MRA, whereas single-modality models exhibited suboptimal performance. Structured report results based on syn-MRA images indicated that the MIX model syn-MRA developed in this study has potential for cerebrovascular screening.

Our results showed that compared to single-modality models, the established MIX model-generated images had better image quality metrics, namely higher SSIM, PSNR, and lower RMSE. Different MR modalities (T1W, T2W, FLAIR) can provide complementary anatomical and pathological information. Compared to single-modality models, multi-modality models often achieved better image quality by integrating these information (19).

Syn-MRA images from all models in this study demonstrated higher SNR and CNR compared to real MRA images. This phenomenon had been widely reported in previous MRA generation studies (13, 15, 20). A potential explanation is the suppression and smoothing of background noise signals in syn-MRA images, thereby leading to significantly elevated SNR and CNR. Additionally, syn-MRA exhibited superior vascular signal uniformity over real TOF-MRA. TOF imaging relies on inflow enhancement effects, which are susceptible to signal loss due to variations in blood flow velocity direction and turbulence, particularly at vessel curvature segments. Although the intrinsic mechanisms of deep learning models remain unclear (21), we hypothesize that syn-MRA primarily utilizes the model’s ability to learn intra-image and inter-image structural and signal information for vascular visualization, fundamentally differing from TOF imaging principles and thus being less affected by inflow enhancement limitations. For SNR and CNR measurements, we selected segments with the strongest inflow-enhanced signal (BA) and turbulence-prone areas (ICA-C5). Results showed that syn-MRA from all models significantly reduced the signal intensity gap between BA and ICA-C5, suggesting that generative models hold potential to address vascular signal heterogeneity in TOF-MRA.

**Figure 6:**
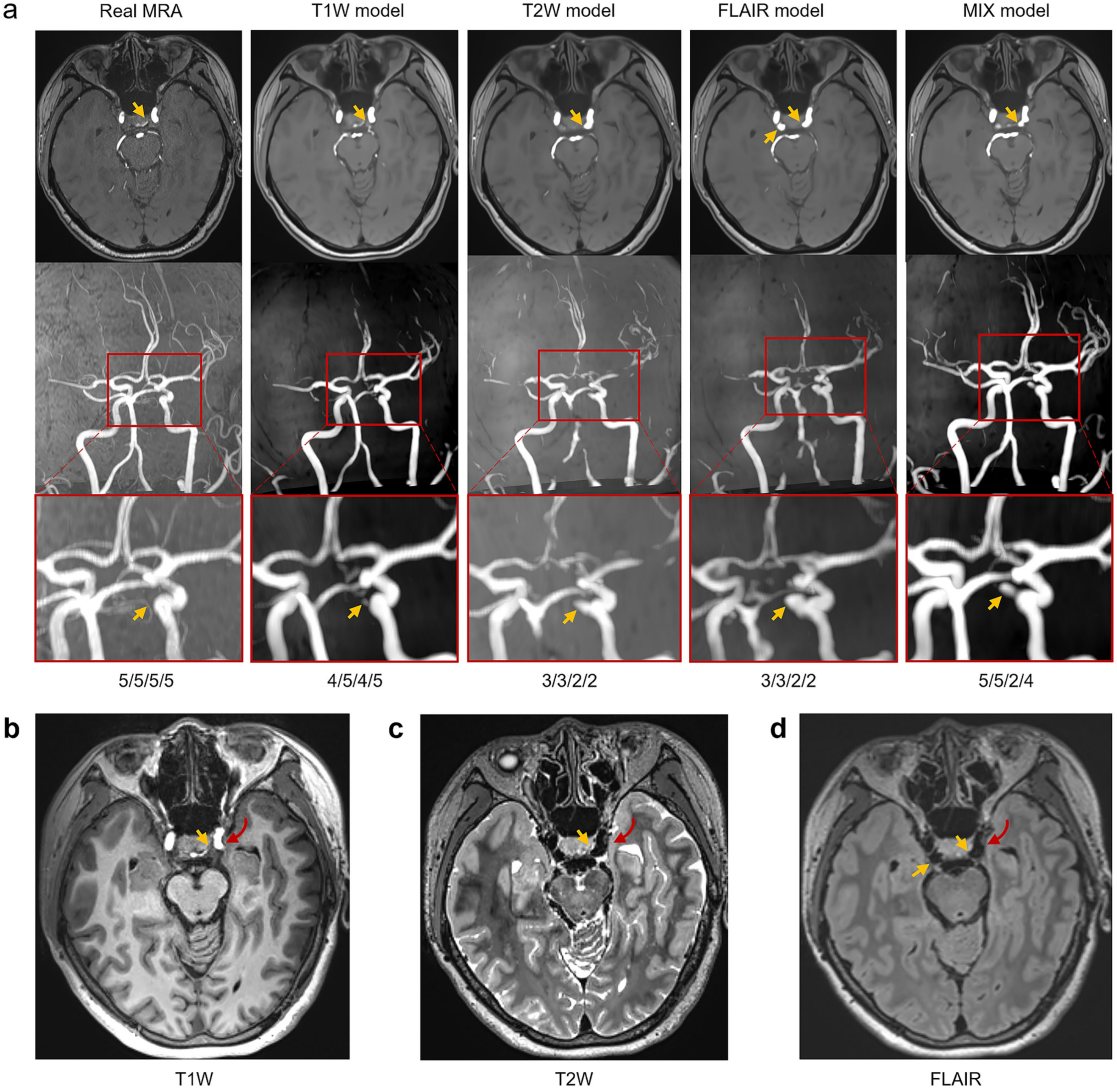
A representative case of cavernous sinus cavity-mediated false-positive aneurysm in synthetic MRA. **(a)** A 56-year-old female with real MRA demonstrating no abnormalities. Synthetic MRA images from T2W, FLAIR, and MIX model depicted a false-positive aneurysm at the left internal carotid artery C4 segment, absent in T1W synthetic MRA (yellow arrow). **(b)** The internal carotid artery demonstrated hyperintensity on T1W source images (red curved arrow), while the location of false-positive aneurysm was hypointense cavernous sinus cavity (yellow arrow). **(c,d)** T2W and FLAIR source images showed flow void signal in both internal carotid artery (red curved arrow) and cavernous sinus cavity (yellow arrow). The red box indicates the magnified image, n/n/n/n means overall image quality/vascular edge sharpness/venous contamination/diagnostic confidence scores. MRA = magnetic resonance angiography, T1W = T1-weighted; T2W = T2-weighted, FLAIR = fluid attenuated inversion recovery.

In the visual quality evaluation of synthetic images, the MIX model-generated images demonstrated optimal overall quality and vascular edge sharpness, reflecting the superiority of the multi-modality model over single-modality approaches, attributable to cumulative multi-contrast and cross-image information integration. Visual quality metrics directly represent radiologists’ perceptual experience (22), and better reflect the consistency between synthetic and real images, which critically influences diagnostic confidence and may impact diagnosis decision-making (23). A key challenge in arterial synthesis is minimizing venous contamination (13), arising from the shared vascular morphology between veins and arteries. While TOF-MRA differentiated arteries via flow velocity difference and venous saturation techniques, generative models lacked dynamic flow velocity information during training, relying solely on static morphological and signal features. This limitation leaded to obvious venous contamination in syn-MRA, particularly in the cavernous sinus region. The anatomical proximity of the cavernous sinus to the internal carotid artery, coupled with structural similarity between sinus cavities and arterial lumens, often resulted in incorrect high signal assignment to sinus structures, accounting for the lower median Likert scores of syn-MRA versus real MRA (median score 3 vs. 5). Critically, severe venous contamination (score ≤ 2) risked misdiagnosis, exemplified by false-positive aneurysms in the cavernous sinus, as illustrated in Figure 6.

Diagnostic performance across all models revealed that the primary challenge lied in maintaining vascular continuity within syn-MRA. Disrupted continuity manifested as elevated false-positive rates for arterial stenosis (>50%), particularly obvious in single-modality models. The FLAIR model exhibited a 55.5% false-positive stenosis rate, compared to 6.8% for the MIX model. The highest failure rate in vascular continuity generation with FLAIR images likely stems from insufficient contrast between vascular structures and surrounding cerebrospinal fluid. T1W images, acquired via gradient recalled echo sequences, demonstrate hyperintense vessels against hypointense cerebrospinal fluid. T2W spin-echo sequences depict flow-void vessels contrasting with hyperintense cerebrospinal fluid. Conversely, FLAIR sequences employ cerebrospinal fluid signal suppression, rendering both vessels and cerebrospinal fluid hypointense. This finding suggests FLAIR sequences may be unsuitable for cranial MRA synthesis. At the vessel level, optimal performance was observed in BA and ICA, whereas other arteries (MCA, ACA, PCA, VA) showed higher false-positive rates. The vascular course direction in these arteries forms a smaller angle with the imaging plane, combined with smaller vessel diameter and increased tortuosity, resulting in diminished arterial signal intensity on original TOF images (24). Such attenuated signals provide weaker contrast and insufficient robust features for the neural network to extract, which may restrict the model’s capacity to accurately reconstruct these specific vascular structures. Beside stenosis detection, single-modality models underperformed in aneurysm and arterial dysplasia identification compared to the multi-modality approach. MRA remains the primary modality for aneurysm screening and surveillance (25). While the MIX model detected all true aneurysms, cavernous sinus interference caused false-positive lesions in syn-MRA. Future researches could integrate attention mechanisms (26) or vascular segmentation model (10) to reduce extravascular signal contamination.

Current findings indicated that single-modality models remained unsuitable for clinical application, whereas the MIX model-based syn-MRA could serve as a screening tool for detecting large intracranial arterial pathologies without requiring additional scan time. However, for aneurysms near the cavernous sinus, TOF-MRA remained necessary for comprehensive evaluation to reduce the risk of misdiagnosed aneurysms.

This study has several limitations: (1) Being a single-center study focusing solely on elderly inpatients, the model’s generalizability requires future validation in larger, multi-center cohorts. (2) As this study focused on 3D modality generative task, model development utilized clinical 3D isotropic datasets. The proposed model was not applicable for 2D images, whose inadequate cross-slice resolution may compromise synthetic MRA quality. Super-resolution networks (27) may provide a viable solution to this challenge. (3) The absence of digital subtraction angiography validation and inherent TOF-MRA imaging limitations may influence diagnostic performance assessments. (4) With a limited sample size of pathological cases and underrepresented disease spectrum, the model’s performance across varied pathological conditions requires additional verification.

## 5 Conclusion

In summary, we developed a deep learning approach capable of generating MRA images based on multi-modality nonvascular images. We evaluated MRA images from geriatric inpatients, demonstrating that syn-MRA achieved image quality comparable to real TOF-MRA, with multi-modality models yielding superior MRA quality compared to single-modality approaches. The syn-MRA generated through our method holds potential for screening intracranial large vessel pathologies and reduces incidental missed diagnosis.

## Data Availability

The raw data supporting the conclusions of this article will be made available by the authors, without undue reservation.
