# Supplementary material for: Utilizing conditional generative adversarial network to generate head MRA based on nonvascular sequences: comparative study of single-modality and multi-modality methods
Source: Front Neurol. 2026 Jul 6;17:1857271. doi: 10.3389/fneur.2026.1857271 (PMC13381828; doi:10.3389/fneur.2026.1857271)
Supplement: Supplementary file 1 [file Supplementary_file_1.DOCX]

Supplementary Material

**Supplementary Material 1**

**Imaging preprocessing**

Owing to the differences in imaging angles and field of view among the four modalities (T1W: Sagittal, 256 mm × 256 mm; T2W: Sagittal, 256 mm × 240 mm; FLAIR: Sagittal, 230 mm × 230 mm; TOF-MRA: Axial, 181 mm × 200 mm), it was necessary to perform image registration for T1W, T2W, and FLAIR images with TOF-MRA images prior to experimentation. In this study, registration was conducted using the built-in registration methods provided by the SimpleITK library.

All 3D volumes were normalized to the range [0, 1] using min-max normalization prior to training. During inference, to accurately compute image metrics, the synthesized MRA images were denormalized back to their original grayscale ranges based on the original TOF-MRA image data. In practical applications, in the absence of real TOF-MRA images, this can be achieved using the average values across the entire dataset.

**Model architecture details**

The model we use is based on conditional generative adversarial network (cGAN). It consists of two independent modules: the generator and the discriminator. These two components work in tandem, constraining and complementing each other to achieve joint optimization. The model enables the generation of data conditioned on specific input information, ensuring greater control over the output and improving relevance to the desired task. This is particularly beneficial for medical imaging, where outputs need to align with specific clinical conditions or modalities. The adversarial training framework helps produce high-quality, realistic results by optimizing the generator to fool the discriminator, encouraging it to capture fine-grained details and realistic textures.

The underlying architecture of the generator is Unet++. It incorporates dense skip connections to improve feature fusion capabilities, thereby enhancing gradient flow and facilitating network training, as well as improving the representation of complex structures. We introduced a convolutional gating mechanism before the Unet++ input, it enables the model to dynamically adjust the importance of different modalities by learning modality-specific weights. Mathematically, the convolutional gating mechanism can be formulated as $I_{gated}=I\odot\sigma\left( Conv\left( I \right) \right)$, where $I$ denotes the concatenated multi-modality input, $\sigma$ represents the Sigmoid activation function, and $\odot$ denotes element-wise multiplication. The discriminator adopts the Patch-based approach, which evaluates local patches of the image rather than the entire image. This design enables more precise capture of fine-grained details, such as textures and edges, reduces computational complexity, enhances training stability, and improves the generalization capability of the generator.

To avoid stripe artifacts along non-imaging planes in 3D images, we utilize 2.5D input, where 5 adjacent 2D slices are used simultaneously during training, and the outputs are averaged based on their overlapping times. To address the clinical preference for maximum intensity projection (MIP) views in vascular diagnosis, we introduce a novel MIP loss function based on Gumbel sampling. In TOF-MRA images, vascular structures appear as hyperintensity regions. The Gumbel sampling mechanism approximates the distribution of hyperintensity, enforcing constraints on clinically significant regions and improving the quality and accuracy of the synthesized vascular features. The computation process is as follows:

First, given a real TOF-MRA images $I_{TOF}\in\mathbb{R}^{N\times W\times H}$, calculate the maximum intensity projection image $I_{mip}$​:

$I_{mip}\left[ i, :, : \right]=max\left( I_{TOF}\left[ 0:i, :, : \right] \right) \in\mathbb{R}^{N\times W\times H}, i\in\left\{ 0,\ldots, N-1 \right\}$,

where N is the number of slices, and W and H denote the width and height of the image.

Then, to capture the distribution of $I_{sync-TOF}$ maximum values $\hat{I}_{mip}\in\mathbb{R}^{N\times W\times H}$ and enable gradient backpropagation, Gumbel random sampling is used to model the maximum values of the synthesized image:

$$\hat{I}_{mip}\left[ i, :, : \right]=\sum_{j=0}^{i} \left( softmax\left( \frac{I_{sync-TOF}\left[ 0:j, :, : \right]+R_{Gumbel}}{T} \right)\cdot I_{sync-TOF}\left[ 0:j, :, : \right] \right)$$

$R_{Gumbel}=-\ln\left( -\ln\left( U+1^{-20} \right)+1^{-20} \right)$,

where $T$ is the temperature coefficient, and $U$ is a random number following the $U\left( 0, 1 \right)$ distribution. Finally, the MIP loss is calculated as follows:

$$f_{MIP}\left( I_{TOF}, I_{sync-TOF} \right)=f_{L1}\left( I_{mip},\hat{I}_{mip} \right)+f_{SSIM}\left( I_{mip},\hat{I}_{mip} \right)$$

where L1 and SSIM are loss functions used to measure similarity.

**Supplementary Material 2**

**Calculation Formula of Quantitative Evaluation**

SSIM, PSNR and RMSE are indicators of the similarity between the original images and the SR-reconstructed images. They are defined as follows:

$$SSIM=\frac{(2\mu_{y}\mu_{y'}+C_{1})(2\sigma_{yy'}+C_{2})}{(\mu_{y}^{2}+\mu_{y'}^{2}+C_{1})(\sigma_{y}^{2}+\sigma_{y'}^{2}+C_{2})}$$

where $\mu_{y}$ and $\mu_{y'}$ represent the mean of the real image $y$ and the synthetic image $y'$ respectively; $\sigma_{y}$ and $\sigma_{y'}$ represent the variance of the image $y$ and $y'$, respectively; $\sigma_{\text{yy'}}$ represents the covariance of images y and $y'$; and $C_{1}$ and $C_{2}$ are constant values used to maintain stability. The SSIM value ranges from -1 to 1, with values closer to 1 indicating better image consistency.

$$PSNR=10\times\text{lo}\text{g}_{10}\left( \frac{{MAX}^{2}}{MSE} \right)$$

Where MAX represents the maximum value of image pixels, MSE represents mean squared error between the pixel values of the synthetic image and the real image. The unit of PSNR is decibels (dB), and the higher the value, the better the image quality.

$$RMSE= \sqrt{\frac{1}{n}\sum_{i=1}^{n} \left( y_{i}-{y'}_{i} \right)^{2}}$$

RMSE measures the standard deviation of the differences between the real image $y$ and the synthetic image $y'$; the smaller it is, the better the result.

$$SNR=\frac{Mean of SI(artery)}{SD of SI(\mathrm{brainstem})}$$

$$CNR=\frac{Mean of SI(artery) - Mean of SI(\mathrm{brainstem})}{SD of SI(\mathrm{brainstem})}$$

Where SD means standard deviation, SI means signal intensity.

**Table S1** Imaging protocol

| **Scanning sequence** | 3D T1W | 3D T2W | 3D FLAIR | 3D TOF-MRA |
| --- | --- | --- | --- | --- |
| **TE/TR (ms)** | 2.56/3000 | 563/3200 | 387/5000 | 3.43/21 |
| **Excitation flip angle (degree)** | 7 | 120 | 120 | 18 |
| **Field of view (mm^2^)** | 256x256 | 240x256 | 230x230 | 181x200 |
| **Image matrix** | 320x320x208 | 320x300x208 | 256x256x192 | 320x275x219 |
| **Slice thickness (mm)** | 0.8 | 0.8 | 0.9 | 0.65 |
| **Pixel spacing (mm^3^)** | 0.8x0.8x0.8 | 0.8x0.8x0.8 | 0.9x0.9x0.9 | 0.625x0.625x0.65 |
| **Acquisition time (minutes)** | 5:31 | 5:49 | 5:30 | 5:27 |

Abbreviations: TE=echo time, TR= repetition time, T1W= T1-weighted, T2W= T2-weighted, FLAIR=fluid-attenuated inversion recovery, TOF-MRA= time of flight magnetic resonance angiography.

**Table S2** Visual quality scoring and diagnostic confidence scoring (5-point Likert scale)

| **Score** | **Grade** | **Details** |
| --- | --- | --- |
| **Overall image quality** | | |
| **5** | **Excellent** | Excellent contrast, no artifacts, and easily identifiable for all main vessel segments. |
| **4** | **Good** | Satisfactory contrast, minor artifacts, all main vessels clearly visualized but image quality somewhat reduced. |
| **3** | **Moderate** | Moderate contrast, modest artifacts, but all main vessels recognition remain feasible. |
| **2** | **Poor** | Poor contrast, significant artifacts, indistinct vessel structures, and inadequate clarity to assess vascular abnormalities. |
| **1** | **Nondiagnostic** | Severe artifacts, no distinct vascular structures visible. |
| **Vascular edge sharpness** | | |
| **5** | **Satisfyingly sharp** | The vascular boundaries are well-defined with high clarity. |
| **4** | **Moderately sharp** | The vascular boundaries are visible but slightly blurred. |
| **3** | **A little sharp** | The vascular boundaries are visible yet markedly blurred. |
| **2** | **Not sharp** | The vascular boundaries are indistinct. |
| **1** | **Nondiagnostic** | No distinct vascular structures visible. |
| **Venous contamination** | | |
| **5** | **None** | No venous structures are visualized. |
| **4** | **Mild** | Venous structures are visualized, but they can be distinctly differentiated from arterial vessels. |
| **3** | **Moderate** | Visualized venous structures close to the arterial vessels, yet they remain distinguishable. |
| **2** | **Severe** | Visualized venous structures close proximity to arterial structures, which impacts the diagnosis. |
| **1** | **Nondiagnostic** | No distinct vascular structures visible. |
| **Diagnostic Confidence** | | |
| **5** | **Definite** | Clear abnormal vascular structures, definitive diagnosis. |
| **4** | **Probable** | Suboptimal image quality, a few artifacts and venous influences, but still sufficient for diagnosis. |
| **3** | **Possible** | Suboptimal image quality, prominent artifacts and venous influences, diagnostic confidence somewhat reduced. |
| **2** | **Uncertain** | Poor image quality, severe artifacts and venous influences, precluding a definitive diagnosis. |
| **1** | **Nondiagnostic** | No distinct vascular structures visible. |

**Table S3** Baseline characteristics of patients

|  | Training set | Validation set | Test set |
| --- | --- | --- | --- |
| No. of patients | 98 | 14 | 28 |
| Age | 68±8 | 65±8 | 71±8 |
| Male | 36(36.7) | 6(42.9) | 11(39.3) |
| Radiological diagnosis | | | |
| Arterial stenosis (＞50%) | … | … | 2(7.1) |
| Aneurysm | … | … | 3(10.7) |
| Arterial dysplasia | … | … | 9(32.1) |
| Normal artery | … | … | 15(53.6) |

Note: The data in the table are presented in mean ± standard deviation or case (%).

**Table S4** Ablation study evaluating the impacts of input modalities and the MIP loss on syn-MIP-MRA

| Models | MIP-loss | | |  | no-MIP loss | | | |  |
| --- | --- | --- | --- | --- | --- | --- | --- | --- | --- |
|  | SSIM | PSNR | RMSE |  | | SSIM | PSNR | RMSE | |
| T1W | 0.754±0.060 | 25.62±1.91 | 0.054±0.012 |  | | 0.736±0.062 | 24.75±2.01 | 0.057±0.013 | |
| T2W | 0.770±0.058 | 26.16±1.75 | 0.050±0.011 |  | | 0.754±0.062 | 25.13±1.84 | 0.053±0.010 | |
| FLAIR | 0.759±0.056 | 25.67±1.72 | 0.053±0.011 |  | | 0.742±0.057 | 24.62±1.66 | 0.055±0.011 | |
| T1W+T2W | 0.774±0.063 | 26.13±2.00 | 0.051±0.013 |  | | 0.759±0.059 | 24.94±2.12 | 0.054±0.013 | |
| MIX | **0.785±0.059** | **26.32±1.91** | **0.049±0.012** |  | | **0.762±0.058** | **24.94±1.76** | **0.058±0.012** | |

Note: The results are presented as mean ± standard deviation, and the best measures are highlighted in bold. The higher PSNR and SSIM, the lower the RMSE values indicate better performance. Abbreviations: syn-MIP-MRA = synthetic maximum intensity projection magnetic resonance angiography, T1W= T1-weighted, T2W= T2-weighted, FLAIR= fluid attenuated inversion recovery, SSIM= structural similarity index measure, PSNR = peak signal-to-noise ratio, RMSE= root mean square error.

**Table S5** Interobserver agreements for vascular quantitative analysis and visual quality scoring among real MRA and syn-MRAs

|  | Weighted kappa or intraclass correlation coefficient (95% CI) | | | | |
| --- | --- | --- | --- | --- | --- |
|  | Real MRA | Syn-MRA  (T1W) | Syn-MRA  (T2W) | Syn-MRA  (FLAIR) | Syn-MRA  (MIX) |
| **SNR** | | | | | |
| R-ICA-C5 | 0.975(0.904-0.994） | 0.923(0.721-0.980) | 0.960(0.847-0.990) | 0.945(0.797-0.986) | 0.986(0.943-0.996) |
| L-ICA-C5 | 0.975(0.902-0.994） | 0.956(0.835-0.989) | 0.983(0.931-0.996) | 0.926(0.732-0.981) | 0.990(0.960-0.997) |
| BA | 0.972(0.892-0.993） | 0.784(0.344-0.942) | 0.703(0.175-0.917) | 0.812(0.412-0.950) | 0.965(0.866-0.991) |
| **CNR** | | | | | |
| R-ICA-C5 | 0.980(0.921-0.995） | 0.886(0.608-0.970) | 0.941(0.781-0.985) | 0.931(0.750-0.982) | 0.977(0.912-0.994) |
| L-ICA-C5 | 0.965(0.865-0.991） | 0.938(0.771-0.984) | 0.977(0.911-0.994) | 0.914(0.695-0.978) | 0.986(0.943-0.996) |
| BA | 0.940(0.778-0.875） | 0.696(0.162-0.915) | 0.651(0.080-0.900) | 0.782(0.339-0.941) | 0.947(0.804-0.987) |
| **Visual Quality Scoring** | | | | | |
| Overall image quality | 0.811(0.563-1.059） | 0.911(0.790-1.031) | 0.919(0.808-1.030) | 0.868(0.616-1.120) | 0.875(0.702-1.048) |
| Vascular edge sharpness | 0.81(0.556-1.063) | 0.838(0.662-1.014) | 0.854(0.694-1.013) | 0.785(0.568-1.001) | 0.940(0.831-1.049) |
| Venous contamination | 1(1.000-1.000) | 1(1.000-1.000) | 1(1.000-1.000) | 0.872(0.701-1.043) | 0.955(0.866-1.043) |
| **Diagnostic Confidence** | 0.900(0.709-0.091) | 0.888(0.761-1.015) | 0.928(0.829-1.026) | 0.781(0.370-1.192) | 0.845(0.672-1.018) |

Abbreviations: T1W= T1-weighted, T2W= T2-weighted, FLAIR= fluid attenuated inversion recovery, syn-MRA = synthetic magnetic resonance angiography, SNR= signal-to-noise ratio, CNR = contrast-to-noise ratio, R-ICA = right internal carotid artery, L-ICA = left internal carotid artery, C5 = clinoid segment, BA = basilar artery, CI=confidence internal.


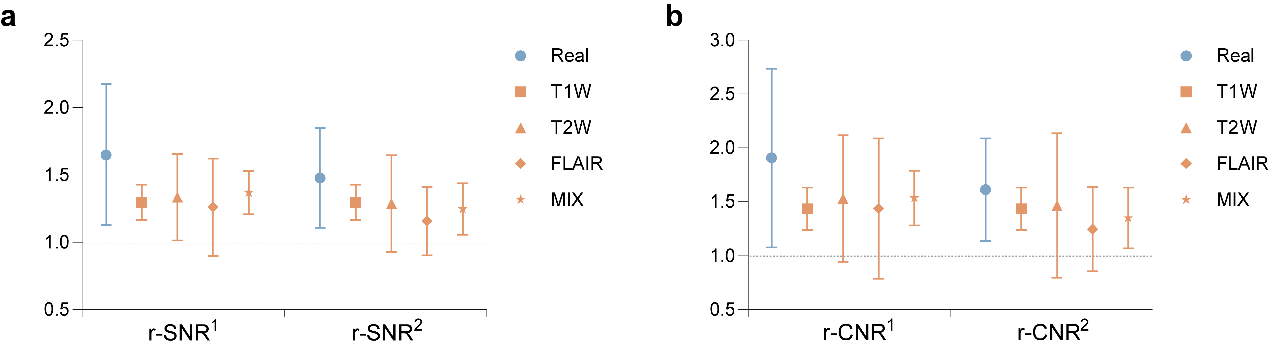


**Figure S1**. Comparison of ratio-signal-to-noise ratio (r-SNR) and ratio-contrast-to-noise ratio (r-CNR) in synthetic MRA images generated by different models. Data are presented as mean ± standard deviation. The mean value closer to the gray reference line (ratio=1) indicates better homogeneity of vascular signal between the basilar artery and the internal carotid artery C5 segment (ICA-C5). Note: r-SNR^1^ and r-CNR^1^ represent the ratio of BA to right ICA-C5; r-SNR^2^ and r-CNR^2^ represent the ratio of BA to left ICA-C5. Abbreviations: T1W= T1-weighted; T2W= T2-weighted; FLAIR= fluid attenuated inversion recovery.

**
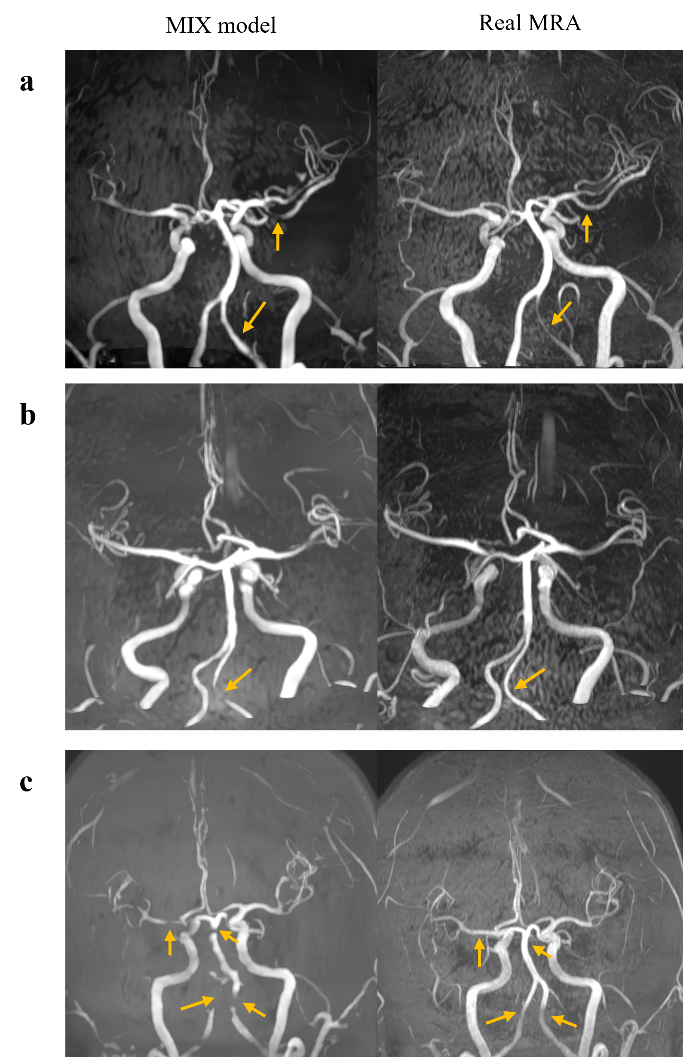
**

**Figure S2**. Failed vascular generation cases in MIX model synthetic MRA (syn-MRA). (a) Syn-MRA depicted a severe stenosis in left middle cerebral artery (false positive) and normal left vertebral artery (false negative), while real MRA revealed a slender left vertebral artery. (b) Syn-MRA demonstrated a local occlusion in left vertebral artery (false positive). (c) Syn-MRA showed occlusions in right middle cerebral artery, basilar artery, and both vertebral arteries (false positive). The bilateral vertebral arteries exhibited insufficient signal intensity on real MRA images. Note: All the aforementioned abnormalities were marked with yellow arrows in images. Abbreviations: MRA = magnetic resonance angiography.
